# Bacterial Meningitis Following Robot-Assisted Radical Prostatectomy After Neoadjuvant Precision Oncology-Directed Therapy: A Case Report

**DOI:** 10.7759/cureus.116726

**Published:** 2026-09-22

**Authors:** Thomas Allen, Gabrielle R Yankelevich, Stephen J Savage, Paul Womble

**Affiliations:** 1 Department of Urology, Medical University of South Carolina, Charleston, USA; 2 Department of Urology, Moffitt Cancer Center, Tampa, USA; 3 Department of Surgery, Ralph H. Johnson Veterans Affairs Health Care System, Charleston, USA

**Keywords:** clinical trial, immunotherapy, meningitis, prostate cancer, pten deficiency

## Abstract

To our knowledge, bacterial meningitis following radical prostatectomy has not been previously reported. We present a 69-year-old male who had phosphatase and tensin homolog (PTEN)-deficient high-risk locoregional prostate cancer and received neoadjuvant leuprolide, abiraterone, prednisone, and capivasertib in the Single-Arm Phase II Study of Neoadjuvant Intensified Androgen Deprivation in Combination with protein kinase B (AKT) Inhibition (SNARE) trial and developed bacterial meningitis following robot-assisted radical prostatectomy. Diagnosis was established by cerebrospinal fluid analysis demonstrating leukocytosis and markedly elevated protein, although cultures were negative following prior antibiotic exposure. Though capivasertib is not considered overtly immunosuppressive, it is possible that a combination of androgen deprivation, low-dose steroids, alcohol use disorder, and surgical intervention resulted in a permissive immunologic environment for infection. This represents, to our knowledge, the first documented case of bacterial meningitis arising after radical prostatectomy. The relationship to his neoadjuvant therapy remains unclear.

## Introduction

Prostate cancer is the second most common cause of death in men in the United States [1]. Radical prostatectomy with pelvic lymph node dissection is a primary surgical treatment for high-risk localized prostate cancer, most commonly performed using a robot-assisted laparoscopic approach [2]. Tumor suppressor mutations, such as phosphatase and tensin homolog (PTEN) loss, have been associated with poorer prognosis in prostate cancer [3]. The Single-Arm Phase II Study of Neoadjuvant Intensified Androgen Deprivation in Combination with protein kinase B (AKT) Inhibition (SNARE) trial (NCT05593497) is an ongoing neoadjuvant phase II study utilizing leuprolide, abiraterone, and prednisone, with the AKT inhibitor capivasertib, which specifically addresses the PTEN mutation, prior to prostatectomy in patients with PTEN-deficient disease [4].

Bacterial meningitis following urologic procedures is exceedingly rare. Prior reports are confined to cases arising after transrectal prostate biopsy [5]. To our knowledge, no case of bacterial meningitis following radical prostatectomy has been reported. We performed a literature search of PubMed and Google Scholar using combinations of the following search terms: “radical prostatectomy,” “prostatectomy,” “robot-assisted radical prostatectomy,” “bacterial meningitis,” “meningitis,” and “meningitis.” We also reviewed the reference lists of relevant articles and case reports without prior reporting. Here, we describe a patient receiving neoadjuvant androgen deprivation therapy (ADT) and capivasertib who developed bacterial meningitis following prostatectomy and discuss factors that could be related to the development of this previously unreported complication.

## Case presentation

A 69-year-old male with a past medical history significant for paroxysmal atrial fibrillation without anticoagulation, asthma, chronic obstructive pulmonary disease, gastroesophageal reflux disease, benign lymphocytosis under hematology surveillance, obstructive sleep apnea, anxiety, and alcohol use disorder with normal liver function tests (LFTs), who reported he was abstinent for at least six months, was diagnosed with high-risk prostate cancer. He had an elevated prostate-specific antigen (PSA) of 13.44 ng/mL (reference range 1-4 ng/mL) and underwent a pre-biopsy MRI showing a 40cc gland with a Prostate Imaging Reporting and Data System (PI-RADS) 5 lesion in the left posterior peripheral zone of the prostate with capsular abutment and left seminal vesicle invasion (Figure 1). The biopsy was notable for Gleason Grade Group (GG) 5 at the MRI target lesion, with a total of 14/20 cores positive (GG5 5/20 cores with 6-10% pattern 5, GG3 6/20 cores with 71-80% pattern 4, GG2 2/20 cores with 31-40% pattern 4, GG1 1/20 cores). He underwent a prostate-specific membrane antigen (PSMA)-piflufolastat F‑18 positron emission tomography (PET) scan thereafter, showing increased uptake in the prostate, left seminal vesicle, and right pelvic lymph nodes measuring 0.82 cm (Figures 2A-2C).

**Figure 1:**
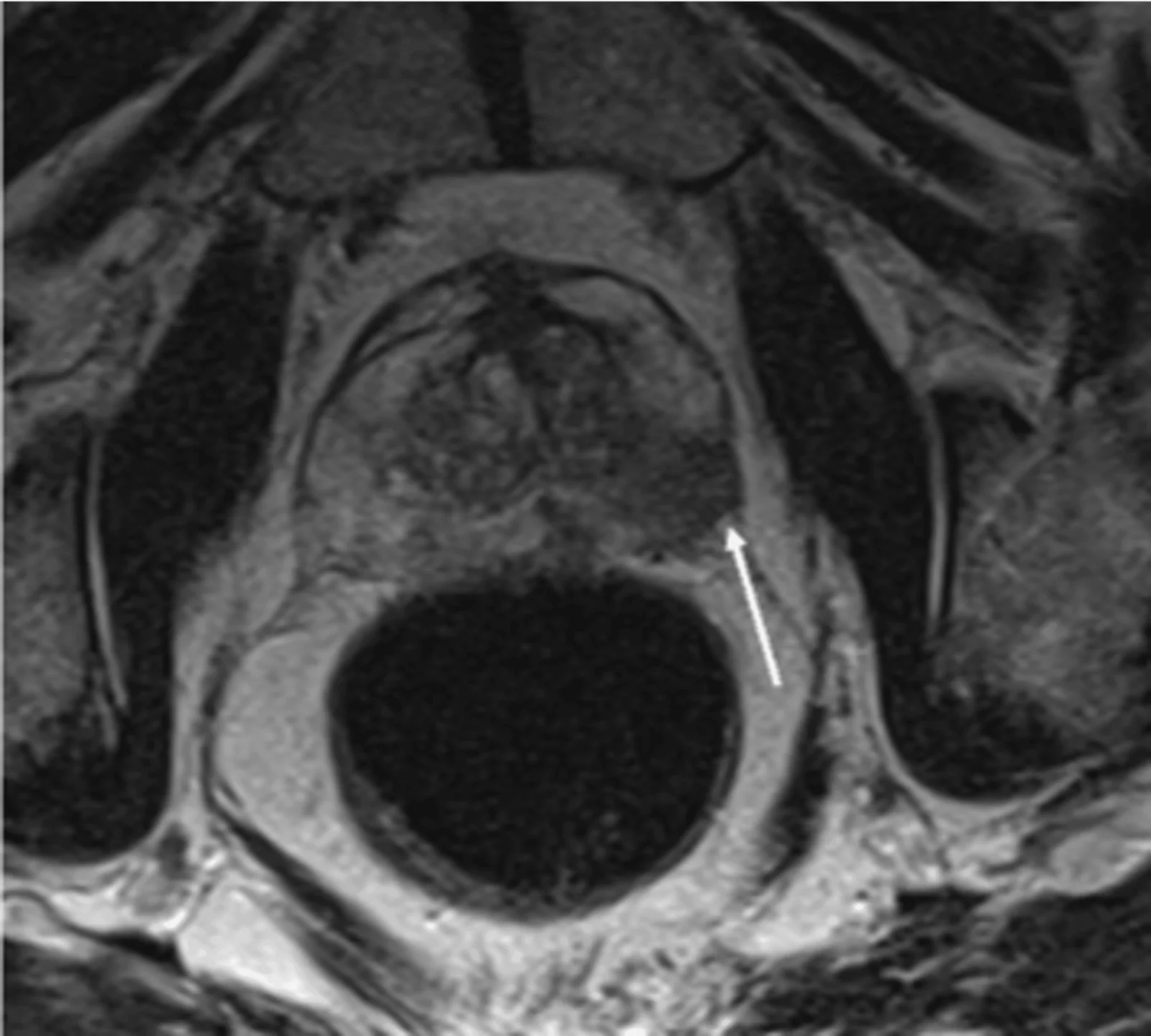
T2-weighted MRI of the prostate showing a 7-mm PI-RADS 5 lesion in the left posterior peripheral zone with capsular abutment (white arrow). PI-RADS: Prostate Imaging Reporting and Data System

**Figure 2:**
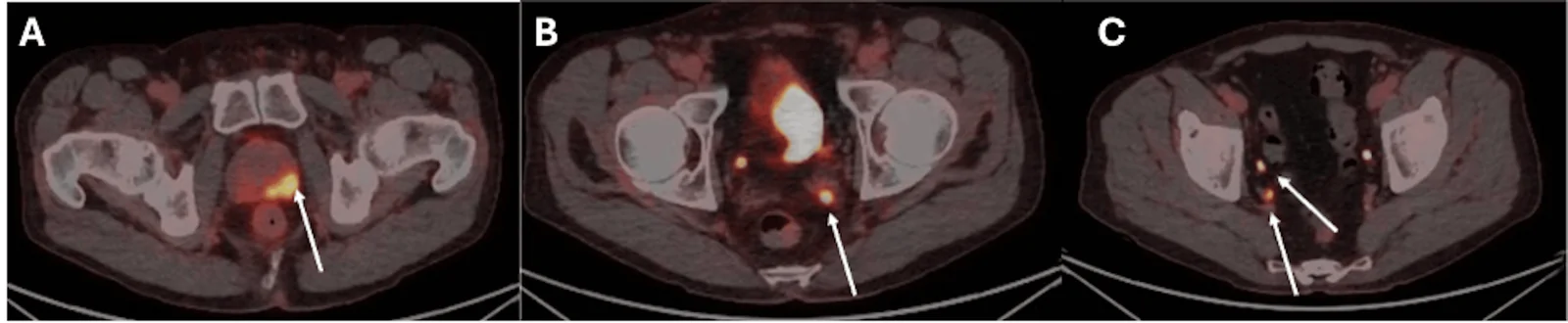
Prostate-specific membrane antigen positron emission tomography (PSMA-PET) imaging. (A) Arrow indicates increased uptake in the left prostate. (B) Arrow indicates increased uptake in the left seminal vesicle. (C) Arrows indicate increased uptake in the right pelvic lymph nodes.

His prostate biopsy was stained for PTEN, showing < 10% expression; therefore, he was offered and enrolled in the SNARE trial. As per study protocol, he received four weeks of neoadjuvant leuprolide, abiraterone, and prednisone, followed by a dedicated research biopsy of the tumor to be used for correlative research, with completion of 16 weeks of the above therapy with the addition of the AKT inhibitor capivasertib. He subsequently presented for robot-assisted radical prostatectomy with pelvic lymph node dissection, in accordance with study protocol. The operative course was uncomplicated. He was monitored postoperatively with a Clinical Institute Withdrawal Assessment for Alcohol (CIWA) protocol [6] given his history of alcohol use disorder, scoring 0 throughout his course, and was discharged on postoperative day 1 following an uneventful recovery.

He presented to the emergency department on postoperative day 5, reporting bothersome scrotal swelling to the emergency department team; however, when evaluated by urology, he stated that his scrotum was not bothering him and that the reason for his visit was a headache. Given the conflicting histories, concern for substance intoxication was raised. Physical examination of the scrotum was consistent with overlying skin cellulitis, so he was given ceftriaxone in the emergency department and discharged on a course of trimethoprim-sulfamethoxazole with return precautions. He reported resolution of his headache prior to discharge.

He returned to the emergency department on postoperative day 6 with a persistent headache and poor appetite. He again provided conflicting accounts to the emergency department and urology teams. Urine drug screen and serum alcohol level were obtained and returned negative. CT of the head without contrast was performed and demonstrated no acute intracranial findings. Admission to the medicine service for altered mental status was discussed with the emergency department team; however, the patient reported he felt better and was again discharged from the emergency department.

On postoperative day 7, he presented to the urology clinic for his planned catheter removal. During the clinic visit, he developed emesis and was directed to the emergency department. At that time, he was febrile to 104°F, tachycardic in the 130s, normotensive, with unlabored breathing and normal oxygen saturation on room air. An infectious workup was initiated, and he was empirically started on vancomycin and piperacillin-tazobactam. Three liters of intravenous fluid were administered, with normalization of his heart rate, and acetaminophen normalized his temperature. He was admitted to the urology service for concern for sepsis. That evening, he developed new-onset photophobia, raising concern for meningitis. A B-type natriuretic peptide (BNP) obtained at that time was elevated to 1,550 pg/mL and rose to 2,431 pg/mL (reference range < 100 pg/mL) when repeated the following morning. Repeat toxicology screening was negative.

Multidisciplinary teams worked to identify sources and treatment plans. Family and friends visiting the patient raised concerns about substance use, yet the clinical team identified a urine drug screen and serum alcohol level that had been negative on multiple occasions. Ultimately, it was determined that the presentation was consistent with meningitis rather than surgical sepsis. An MRI of the brain with contrast was unremarkable. A bedside lumbar puncture attempted by neurology was unsuccessful, and the patient subsequently underwent a lumbar puncture performed by interventional radiology; however, this was after more than 48 hours of antibiotics. Cerebrospinal fluid (CSF) analysis is shown in Table 1. CSF cultures were ultimately negative, as were blood cultures. Infectious disease and neurology concluded that the CSF profile and clinical scenario were most consistent with bacterial meningitis but remained negative due to antibiotic duration prior to successful lumbar puncture. He was discharged on linezolid, ceftriaxone, and ampicillin for 21 days. His urinary catheter was removed during the admission, and he was able to void spontaneously. The final surgical pathology returned as pT3bN0 with treatment effect. At his three-month surveillance visit, his PSA was undetectable.

**Table 1:** Cerebrospinal fluid (CSF) analysis values.

CSF Component	Patient Value	Reference Range
Red Blood Cell Count	6000 cells/μL	0 cells/μL
White Blood Cell Count	33 cells/μL	0-4 cells/μL
Protein Concentration	>200 mg/dL	15-45 mg/dL
Glucose	64 mg/dL	50-80 mg/dL

## Discussion

It is rare for urologists to encounter meningitis in clinical practice; however, understanding the diagnosis and management is crucial as bacterial meningitis carries significant morbidity and mortality [7]. Meningitis most commonly affects elderly patients 60 years and older and immunocompromised adults [7]. The classic triad of fever, neck stiffness, and altered mental status is present in only 41% of patients [7]. When suspected, empirical ampicillin is recommended for older and immunocompromised patients given the elevated risk of *Listeria monocytogenes* infection [7]. Though the fever was not present until postoperative day 7, our patient showed signs of waxing and waning mental status prior. Moreover, a history of alcohol use disorder is a strong independent risk factor for bacterial meningitis with adverse outcomes [8]. This risk reflects chronic immunological compromise rather than acute intoxication [4]. This is the leading proposed mechanism for our patient’s increased risk for meningitis.

Culture-negative meningitis is well established when antibiotics precede lumbar puncture, as seen in our patient with antibiotic administration starting with ceftriaxone for cellulitis five days before CSF was obtained. Therefore, the diagnosis rested on leukocytosis and markedly elevated protein, which is supported in the literature [9].

PTEN loss, present in 40% to 50% of patients with metastatic castration-resistant prostate cancer, constitutively activates the PI3K/AKT/mTOR axis and is associated with aggressive disease behavior and poor clinical outcomes [10]. The SNARE trial targets this vulnerability with neoadjuvant ADT plus capivasertib (AZD5363), a pan-AKT inhibitor, in patients with PTEN-deficient high-risk localized prostate cancer prior to prostatectomy [11]. In the phase Ib trial, leukopenia occurred in 10.5% of patients receiving capivasertib, introducing immune compromise not accounted for in standard post-prostatectomy risk models [12], although no leukopenia was present in this case.

Recently, the CAPItello-281 phase III trial results were reported in the metastatic hormone-sensitive prostate cancer population where those with PTEN deficiency received capivasertib or placebo in addition to abiraterone, prednisone, and ADT [10]. Their study found that the addition of capivasertib improved progression-free survival [11]. There was no increased risk of urinary tract infection and no reported leukopenia or immunosuppression; however, the capivasertib arm did have 1.2% of patients with diabetic ketoacidosis and underlying infection compared to none in the placebo group [11].

Moreover, alcohol abuse is associated with the risk of infectious disease, including bacterial pneumonia and bacterial meningitis [13]. The reason for this is twofold. Alcohol abuse itself suppresses innate and adaptive immune responses, which causes increased risk for infections [13]. Also, malnutrition is related to chronic alcohol abuse and contributes to immunosuppression [13].

This patient's immunosuppressive burden can be proposed to have contributions from four concurrent mechanisms. At the drug class level, PI3K/AKT/mTOR inhibitors carry an infection odds ratio (OR) of 4.26 (95% CI 1.9 to 9.1) versus other targeted agents, a finding independently replicated at OR 3.55 in a separate phase I cohort [14,15]. However, there was no significant risk of infection in the capivasertib-treated group when compared to placebo in the CAPItello-281 study [11]. Second, ADT independently increases infection risk, with data from over 52,000 patients demonstrating a dose-dependent rise in community-acquired respiratory infections [16]. Third, chronic corticosteroid use is an established risk factor for opportunistic infection [17]. Fourth, alcohol use disorder independently suppresses innate and adaptive immunity and was associated with unfavorable outcomes in a population-based cohort of patients with alcohol-related bacterial meningitis [7]. It is unknown to what extent each factor may have contributed to this clinical scenario, and the local and national study team determined the meningitis to not be causally related to study treatment.

A systematic review identified 13 prior cases of bacterial meningitis following urologic procedures, all after transrectal prostate biopsy and all caused by *Escherichia coli* [5]. The proposed mechanism is hematogenous seeding through the direct vascular connection between the periprostatic and lumbar vertebral venous plexuses, with retrograde flow during periods of elevated intra-abdominal pressure [5]. In our patient, the transabdominal approach excludes direct CNS contamination, and the absence of epidural anesthesia eliminates a known iatrogenic route, leaving hematogenous dissemination as the most plausible mechanism. In a contemporary NSQIP cohort of over 11,800 patients undergoing robot-assisted radical prostatectomy, CNS infection was absent from the reported complication taxonomy [18]. To our knowledge, this is the first reported case of bacterial meningitis following radical prostatectomy of any surgical approach.

## Conclusions

Bacterial meningitis following radical prostatectomy is an exceedingly rare complication with no prior reports in the literature. This case demonstrates that it is important to take into consideration all contributing factors while also paying close attention to any unusual presenting symptomatology. Further evidence in patients receiving multimodal therapies will be necessary to better elucidate relative risk for unusual infectious complications.
